# Targeting endothelial vascular cell adhesion molecule-1 in atherosclerosis: drug discovery and development of vascular cell adhesion molecule-1–directed novel therapeutics

**DOI:** 10.1093/cvr/cvad130

**Published:** 2023-08-18

**Authors:** Jessica R Pickett, Yuao Wu, Lucia F Zacchi, Hang T Ta

**Affiliations:** Queensland Micro- and Nanotechnology Centre (QMNC), Griffith University, West Creek Road, Nathan, QLD 4111, Australia; School of Environment and Science, Griffith University, Kessels Road, Nathan, QLD 4111, Australia; Queensland Micro- and Nanotechnology Centre (QMNC), Griffith University, West Creek Road, Nathan, QLD 4111, Australia; Australian Institute for Bioengineering and Nanotechnology (AIBN), University of Queensland, St. Lucia, QLD 4072, Australia; School of Chemistry and Molecular Biosciences, the University of Queensland, St. Lucia, QLD 4072, Australia; Queensland Micro- and Nanotechnology Centre (QMNC), Griffith University, West Creek Road, Nathan, QLD 4111, Australia; School of Environment and Science, Griffith University, Kessels Road, Nathan, QLD 4111, Australia

**Keywords:** Atherosclerosis, Cardiovascular disease, Inflammation, VCAM-1, Anti-VCAM-1 therapy

## Abstract

Vascular cell adhesion molecule-1 (VCAM-1) has been well established as a critical contributor to atherosclerosis and consequently as an attractive therapeutic target for anti-atherosclerotic drug candidates. Many publications have demonstrated that disrupting the VCAM-1 function blocks monocyte infiltration into the sub-endothelial space, which effectively prevents macrophage maturation and foam cell transformation necessary for atherosclerotic lesion formation. Currently, most VCAM-1-inhibiting drug candidates in pre-clinical and clinical testing do not directly target VCAM-1 itself but rather down-regulate its expression by inhibiting upstream cytokines and transcriptional regulators. However, the pleiotropic nature of these regulators within innate immunity means that optimizing dosage to a level that suppresses pathological activity while preserving normal physiological function is extremely challenging and oftentimes infeasible. In recent years, highly specific pharmacological strategies that selectively inhibit VCAM-1 function have emerged, particularly peptide- and antibody-based novel therapeutics. Studies in such VCAM-1–directed therapies so far remain scarce and are limited by the constraints of current experimental atherosclerosis models in accurately representing the complex pathophysiology of the disease. This has prompted the need for a comprehensive review that recounts the evolution of VCAM-1–directed pharmaceuticals and addresses the current challenges in novel anti-atherosclerotic drug development.

## 1. Introduction

**Figure 1:**
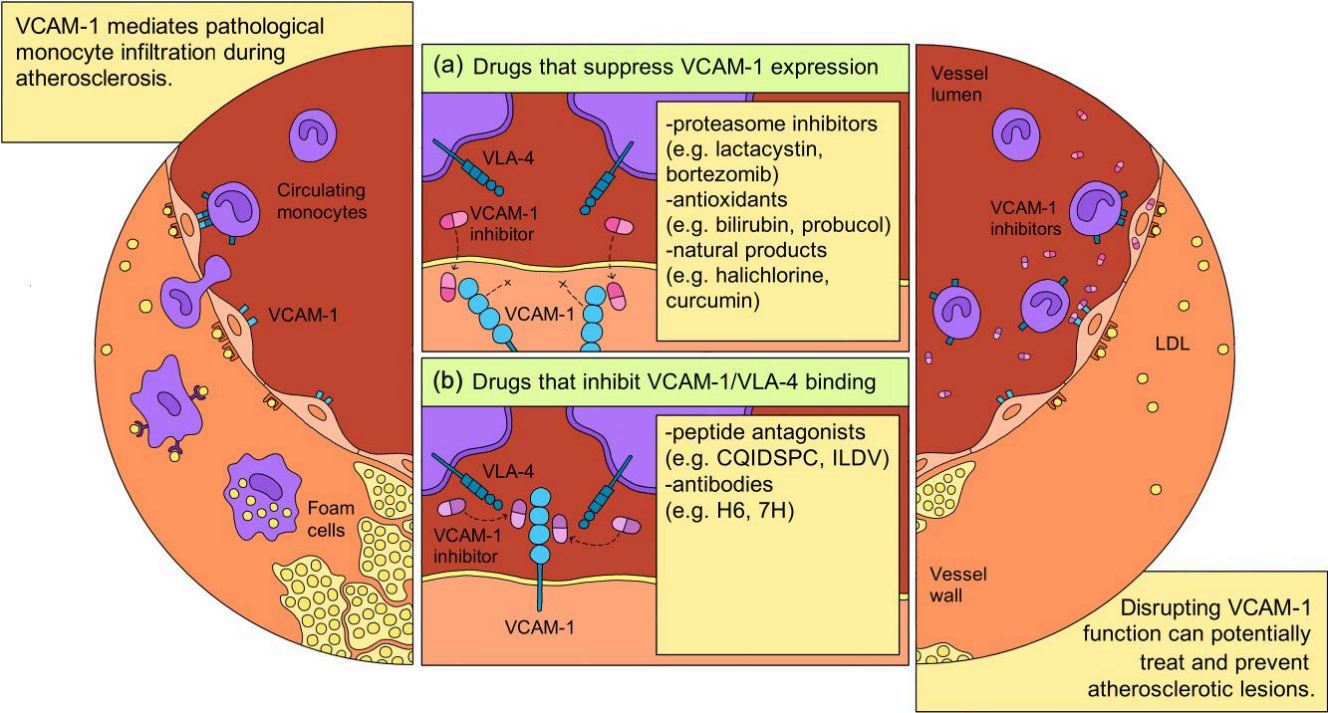
Summarizing the rationale of VCAM-1–directed therapies for the treatment of atherosclerosis. VCAM-1–inhibiting drug candidates can be broadly categorized based on two pharmacological mechanisms: (a) drugs that suppress VCAM-1 expression and (b) drugs that inhibit the VCAM-1/VLA-4 interaction involved in endothelial-monocyte attachment.

Atherosclerosis is an occlusive, inflammatory disease of the vasculature characterized by the build-up of fibro-fatty plaque, or atheroma, within the arteries.^1,2^ Over time, the definition of atherosclerosis has evolved from a relatively simple, passive cholesterol storage condition to a highly complex, chronic inflammatory disease driven by endothelial dysfunction and dysregulated immune cell infiltration.^3,4^ As such, research into novel anti-atherosclerotic therapies has shifted from conventional lipid-lowering strategies to directly addressing molecular targets and pathways involved in vascular inflammation.^5–9^ Vascular cell adhesion molecule-1 (VCAM-1) has emerged as a promising drug target for treating atherosclerosis and associated cardiovascular disease (CVD) due to its involvement in monocyte recruitment and selective up-regulation on atheroprone regions of the vascular endothelium.^10^ This literature review aims to describe the development and progression of anti-atherosclerotic drugs that act by inhibiting VCAM-1 activity, either indirectly through modulating upstream mediators and intermediaries or by directly blocking VCAM-1–mediated interactions (*Figure 1*). The rapid acceleration of atherosclerotic research over the last several years has prompted the need for a comprehensive review that discusses recent advances in VCAM-1–directed pharmaceuticals and addresses arising challenges in the pre-clinical testing of novel therapeutics.

## 2. Pathophysiology of atherosclerosis

**Figure 2:**
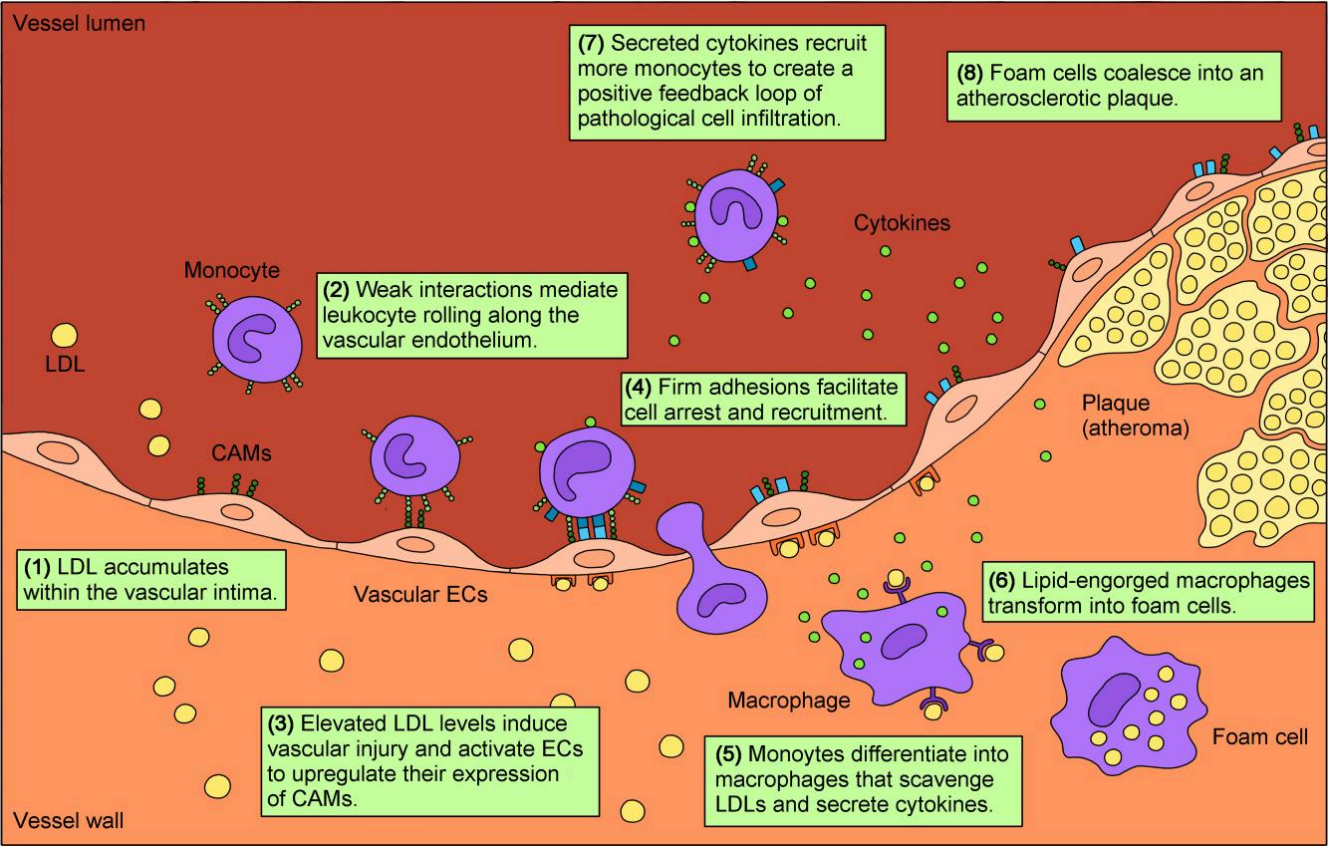
A conceptual diagram illustrating pathological monocyte recruitment during atherosclerosis. The accumulation of LDL infiltrates within the sub-intimal space activates vascular ECs to up-regulate their expression of CAMs. These CAMs mediate firm monocyte–endothelial interactions that facilitate cell adhesion and transmigration across the vascular endothelium. Once within the sub-endothelial space, recruited monocytes differentiate into macrophages, which uptake excess LDL at the expense of transforming into lipid-engorged foam cells. Additionally, macrophages secrete cytokines to establish a positive feedback loop of continual monocyte recruitment and accumulation. Eventually, transformed foam cells amalgamate to form an atheromatous plaque.

Pathological monocyte recruitment is an indispensable process in the initiation and progression of atherosclerotic lesions.^11^ The ability of macrophages to ingest LDL means that regulated monocyte recruitment is a necessary defence against sub-endothelial lipid accumulation. However, chronic endothelial activation during atherosclerosis creates a vicious cycle of constant monocyte influx that drives plaque development and destabilization (*Figure 2*).^12,13^ Activated endothelial cells (ECs) up-regulate their surface expression of cell adhesion molecules (CAMs), including E-selectin, P-selectin, inter-cellular adhesion molecule-1 (ICAM-1), and VCAM-1.^14^ While E-selectin and P-selectin support initial monocyte capture and rolling on the vascular endothelium, firmer attachments mediated by VCAM-1 and ICAM-1 expedite monocyte arrest and transmigration across the vessel wall.^15,16^ Monocytic maturation factors secreted by ECs trigger the differentiation of recruited monocytes into macrophages, which are able to uptake and phagocytose oxidized LDL at the expense of transforming into the lipid-engorged foam cells that constitute atheromatous plaque.^17–20^ Lesional macrophages also contribute to endothelial dysfunction by secreting the inflammatory cytokine interleukin-1β (IL-1β), effectively creating a self-perpetuating positive feedback loop that exacerbates plaque progression.^21^ In addition to VCAM-1–mediated cell recruitment, local macrophage proliferation and vascular smooth muscle cell trans-differentiation are also partially responsible for supplying cells to the growing plaque.^13,22,23^ With no route of exit, continuing macrophage accumulation and apoptosis eventually coalesces into an acellular necrotic core within the plaque, disrupting the normal architecture of the vascular wall to establish a characteristic atherosclerotic lesion.^24^

## 3. VCAM-1: a promising anti-adhesion target for atherosclerosis

**Figure 3:**
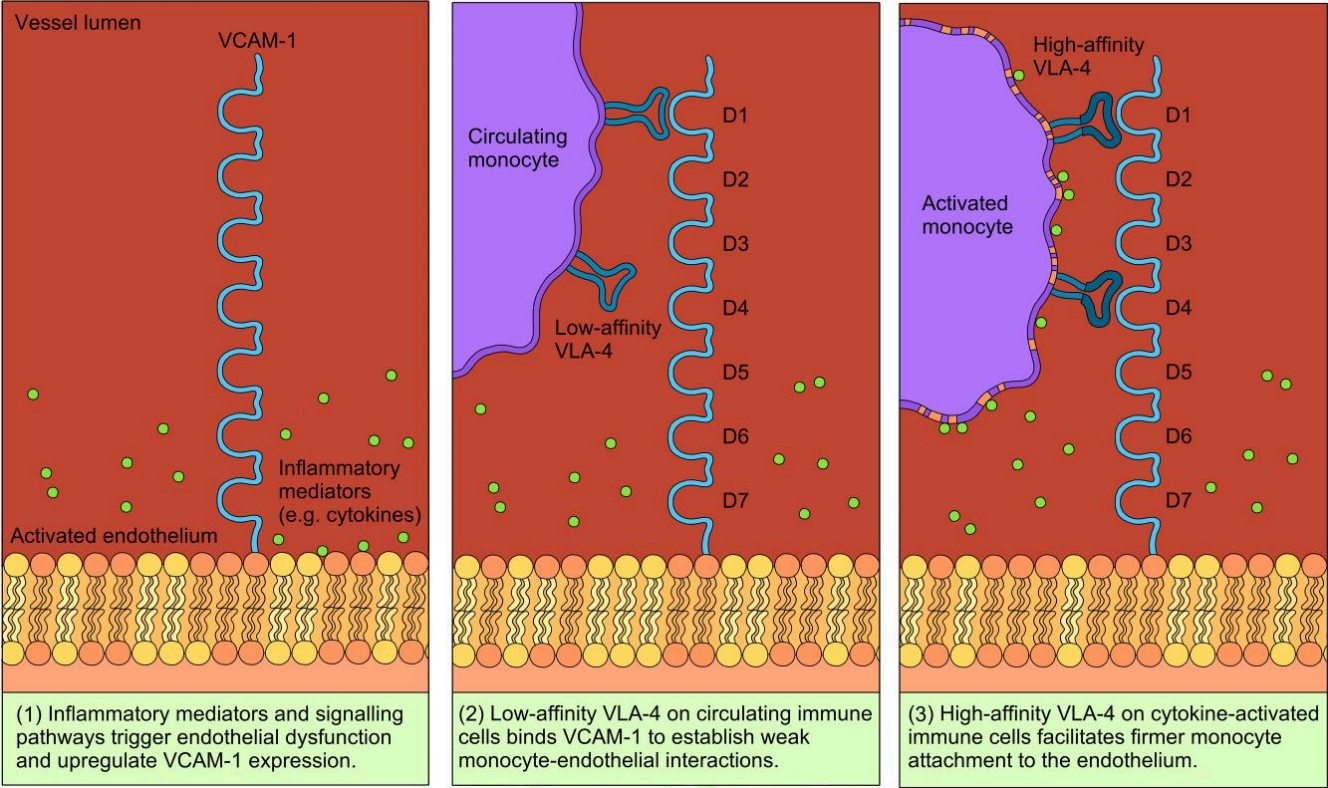
Binding interactions between VCAM-1 on vascular ECs and VLA-4 integrin on circulating monocytes during vascular inflammation. (1) VCAM-1 expression is induced on activated ECs in response to pro-inflammatory conditions. (2) Circulating monocytes express the low-affinity conformation of VLA-4, which can only bind to domain 1 of VCAM-1. The resultant VCAM-1/VLA-4 interaction mediates the weak binding associated with rolling leucocyte adhesion. (3) The high-affinity conformation of VLA-4 is activated in response to inflammation and can bind both Domains 1 and 4 of VCAM-1. This stronger version of the VCAM-1/VLA-4 interaction is responsible for the firm monocyte-endothelial adhesions necessary for monocyte arrest and transmigration.

VCAM-1 is a cytokine-inducible surface glycoprotein expressed predominantly on the luminal and lateral surfaces of activated ECs during inflammation.^25^ It is a member of the immunoglobulin superfamily (IgSF)—along with ICAMs, platelet/EC adhesion molecule-1, and mucosal addressin CAMs—all of which act as receptors for their corresponding integrin ligands on circulating leucocytes.^26^ Human VCAM-1 exists in two alternatively spliced isoforms, a six IgSF domain form and a more dominantly expressed seven-domain form.^27,28^ Domains 1–3 of the seven-domain VCAM-1 isoform are highly homologous to Domains 4–6, with Domains 1 and 4 both carrying the integrin-binding Ile-Asp-Ser-Pro-Leu (IDSPL) amino acid sequence recognized by very late antigen-4 (VLA-4) integrin during leucocyte adhesion.^27,29,30^ However, while the addition of glutamine to this motif to make Gln-Ile-Asp-Ser-Pro-Leu amino acid sequence in Domain 1 allows binding to non-activated VLA-4 under normal cell conditions, the IDSPL region in Domain 4 can only bind to high-affinity VLA-4, which requires prior activation by divalent cations or chemokines (*Figure 3*).^31^ The immunoregulatory role of VCAM-1 in monocyte infiltration, coupled with its minimal involvement in normal leucocyte trafficking, has therefore marked it as an auspicious therapeutic target for multiple inflammatory diseases, including atherosclerosis.

### 3.1 Molecular mechanisms of VCAM-1 signalling in leucocyte transmigrations

**Figure 4:**
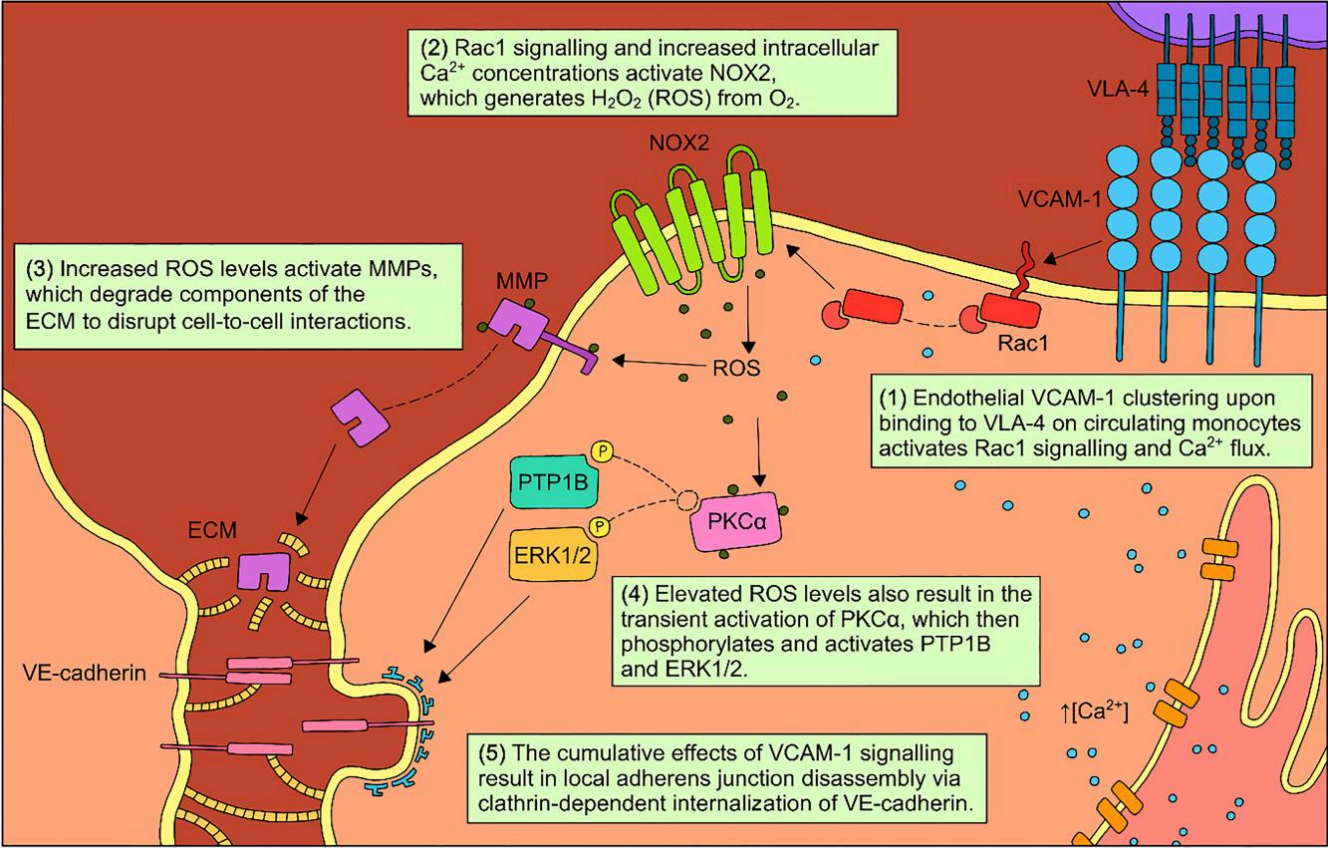
Intra-cellular signalling pathways involved in VCAM-1–mediated transmigration across the vascular endothelium. Clustering interactions between VCAM-1 on activated ECs and VLA-4 on circulating monocytes initiate Ca^2+^ flux and Rac1 activation, both of which consequently activate NOX2. NOX2 generates H_2_O_2_ from O_2_ to activate MMPs and PKCα, which activates PTP1B and ERK1/2. PTP1B and ERK1/2 in conjunction stimulate the disassembly of VE-cadherin from adherens junctions. The cumulative effect of these molecular pathways induces localized weakening of cell-to-cell junctions and gap formation to facilitate cell diapedesis from the vessel lumen into the sub-endothelial space.

The physiological bridge between cell adhesion and transmigration revolves around a network of VCAM-1–dependent signalling pathways that co-ordinate para-cellular transport through cytoskeletal rearrangement and cell-to-cell junctional weakening (*Figure 4*).^32^ VCAM-1 cross-linking during monocyte-endothelial attachment triggers intra-cellular calcium (Ca^2+^) flux and Rac1 guanosine triphosphatase signalling, both of which activate NADPH oxidase 2 (NOX2).^33–36^ NOX2 generates hydrogen peroxide (H_2_O_2_) from oxygen (O_2_), and the resultant increase in intra-cellular reactive oxygen species (ROS) markedly affects local signal transduction.^37,38^ VCAM-1–dependent ROS signalling rapidly activates matrix metalloproteinases (MMPs), which work to disrupt cell-to-cell adhesions by degrading the extracellular matrix and protein components of local adherens junctions.^39^ High intra-cellular ROS levels also result in the direct oxidation and transient activation of protein kinase Cα (PKCα), which phosphorylates and activates EC protein tyrosine phosphatase 1B (PTP1B) and extracellular signal-regulated kinases 1 and 2 (ERK1/2).^40–42^ The cumulative effects of these pathways lead to the downstream phosphorylation and clathrin-dependent internalization of vascular endothelial (VE)-cadherin, which promotes the ‘loosening’ of adherens junctional structures for para-cellular transport across the vascular endothelium.^43,44^

While it is a distinct process from the VCAM-1–mediated internalization of VE-cadherin from adherens junctions, it is also important to acknowledge the role of the lateral border recycling compartment (LBRC) in facilitating leucocyte passage across the endothelial barrier. The LBRC is an inter-connected reticulum contiguous with the plasma membrane that engages and surrounds the transmigrating cell with VE-cadherin–excluding membrane during diapedesis.^45^ This ‘targeted recycling’ of LBRC membrane is hypothesized to push aside VE-cadherin from the cell-to-cell junction and provide the leucocyte with a convenient, adhesion molecule-lined pathway for migration without requiring EC retraction.^46,47^ Although the exact molecular mechanisms are yet to be fully elucidated, VCAM-1 signalling through NOX2, MMPs, PKCα, and PTP1B has been well-established as a pre-requisite process for cell migration in several inflammatory disease pathologies and cancer cell metastasis.^10,48–52^

### 3.2 Characterizing the role of VCAM-1 in atherosclerosis

The role of VCAM-1 in endothelial dysfunction and leucocyte infiltration during vascular inflammation has established VCAM-1 as a critical contributor to the pathogenesis of atherosclerosis.^10,48^ Several studies have directly observed elevated VCAM-1 expression at sites of atherosclerosis, supporting its role in the progression and development of atheromatous lesions through cell arrest and extravasation.^53–57^ Initially, it was ambiguous as to whether VCAM-1 was simply a biomarker of atherogenesis or actually had a causal role in the pathophysiology of the disease.^10^ To provide definitive evidence for the functional role of VCAM-1 in atherosclerosis, Cybulsky *et al.*^58^ generated homozygous VCAM-1 domain 4-deficient (*VCAM1^D4D^*) mice and crossed them with LDL receptor-deficient (*LDLR^−/−^*) mice to create a *VCAM^D4D/D4D^/LDLR^−/−^* mouse line. These *VCAM^D4D/D4D^/LDLR^−/−^* mice demonstrated a degree of atheroresistance stronger than that of plain *LDLR^−/−^* mice, implicating the functional significance of VCAM-1 in atherosclerosis. After properly establishing the role of VCAM-1 in atherogenesis as a mediator of pathological monocyte infiltration,^59–62^ novel therapeutics blocking VCAM-1 function have emerged as an attractive strategy for treating atherosclerosis. The remainder of this review goes on to summarize the existing evidence and current developments supporting the anti-atherogenic potential of several novel therapeutics that interfere with VCAM-1 activity (listed in *Table 1*). The efficacy and limitations of each therapeutics were also included in the Table.

**Table 1:** Summary of efficacy and limitations of notable novel VCAM-1 inhibitors listed in this review

Class	Therapeutic	Efficacy	Limitation(s)	Source(s)
PIs	MG-101	*In vitro:* Blocks IL-1β-induced VCAM-1 activity in ECV304 cells (10–100 µM)	Efficacy has not been assessed *in vivo* or in clinical studies	Cobb *et al.*^63^
		*In vitro:* Blocks NF-κB activation and VCAM-1 mRNA accumulation in angiotensin II-stimulated RASMCs (100 µM)		Tummala *et al.*^64^
	MG-132	*In vitro:* Prevents neutrophil migration in TNF-α–stimulated ECs (20 µM)	Efficacy has not been assessed *in vivo* or in clinical studies	Allport *et al.*^65^
		*In vitro:* Blocks NF-κB-induced VCAM-1 expression in TNF-α–stimulated HUVECs (20 µM). Reduces lymphocyte adhesion to TNF-α–stimulated HUVEC monolayers and blocks transmigration (50 µM)		Read *et al.*^66^
	Lactacystin	*In vitro:* Suppresses VCAM-1 surface expression in TNF-α-stimulated HUVECs. Reduces adhesion of human neutrophils to HUVECs and blocks transmigration (20 µM)	Efficacy has not been assessed in clinical studies. Chronic treatment has demonstrated hypertensive effects *in vivo*^67^	Allport *et al.*^65^
		*In vitro:* Inhibits vascular smooth muscle cell proliferation and migration (10–20 µM) *In vivo:* Inhibits neo-intimal formation in rat carotid vessels (40 µM)		Barringhaus *et al*.^68^
		*In vitro:* Suppresses surface expression of VCAM-1 and inhibits HL60 myeloid cell adhesion in TNF-α and IL-1β-stimulated HUVECs (20 µM)		Dagia and Goetz^69^
		*In vitro:* Blocks NF-κB activation and VCAM-1 mRNA accumulation in angiotensin II-stimulated RASMCs (100 µM)		Tummala *et al.*^64^
	Bortezomib	*In vivo:* Reduces VCAM-1 expression, but not atherosclerotic lesion area in Wistar rats (50 µg/kg/day)	Conflicting results between studies. Adverse CVD events have been observed in patients receiving bortezomib^70,71^	Ismawati *et al.*^72^
		*In vitro:* Reduces VCAM-1 expression both alone and with dimethyl fumarate (5 ng/mL). Reduces leucocyte rolling and adhesion on TNF-α–stimulated ECs with dimethyl fumarate (10 ng/mL)		Hund *et al*.^69^
		*In vivo:* Decreases VCAM-1 expression, atherosclerotic lesion size, macrophage infiltration, and vascular superoxide production in *LDLR^−/−^* mice (50 µg/kg)		Wilck *et al*.^73^
Antioxidants	PDTC	*In vitro:* Selectively inhibits VCAM-1 induction in IL-1β– and LPS-stimulated HUVECs (50 µM). Reduces MOLT-4 cell adhesion to TNF-α–stimulated HUVECs (50 µM)	Efficacy has not been assessed *in vivo* or in clinical studies.	Marui *et al*.^74^
	NAC	*In vitro:* Inhibits IL-1β induction of VCAM-1 mRNA accumulation in HUVECs (30 mM)	Efficacy has not been assessed in clinical studies	Marui *et al.*^74^
		*In vivo:* Decreases plaque distribution and foam cell accumulation in *ApoE^−/−^* mice (300 mg/kg/day)		Zhenxiao *et al.*^75^
	Bilirubin	*In vitro:* Inhibits leucocyte migration across mHEv monolayers without affecting adhesion or VCAM-1 expression (10–20 µM) *In vivo:* Reduces lymphocyte and eosinophil infiltration into the airways of ovalbumin-treated mice (30 mg/kg)	Efficacy has not been assessed in clinical studies	Keshavan *et al.*^76^
		*In vitro:* Inhibits TNF-α–induced VCAM-1 expression and endothelial adhesion in HUVECs and H5V cells (15–30 nM)		Mazzone *et al.*^77^
		*In vitro:* Inhibits THP-1 monocyte migration across TNF-α–stimulated HUVECs (10–20 µM, 120 min). Supresses ROS production after VCAM-1 activation (20 µM)		Vogel *et al.*^78^
	Probucol	*In vivo:* Significantly lowers VCAM-1 expression and reduces presence of intimal macrophages in aortas of *LDLR^−/−^* Watanabe heritable hyperlipidaemic rabbits (1% w/w)	Clinical intervention studies failed to observe a statistically significant improvement with probucol treatment compared to other LLTs^79^	Fruebis *et al.*^80^
		*In vivo:* Supresses VCAM-1 expression and LDL oxidation in Watanabe heritable hyperlipidaemic rabbits (1% w/w)		Murayama *et al.*^81^
		*In vivo:* Significantly decreases plaque area and VCAM-1 expression in *ApoE^−/−^* mice		Wu *et al.*^82^
		*Clinical trial:* Decreases incidence of cerebrovascular and CVD events, but not to a statistically significant extent (500 mg/day)		Yamashita *et al.*^79^
		*In vitro:* Reduces VCAM-1 mRNA surface expression in TNF-α– and IL-1β–stimulated HUVECs. Decreases leucocyte adhesion to cytokine-stimulated HUVECs (50 µM)		Zapolska-Downar *et al.*^83^
	Succinobucol	*In vitro:* Inhibits VCAM-1 expression in TNF-α–stimulated ECs (0.5–10 µM)	Clinical intervention studies failed to observe a statistically significant improvement with succinobucol compared to placebo^84^	Kunsch *et al.*^85^
		*In vivo:* Reduces atherosclerosis in *LDLR^−/−^* and *ApoE^−/−^* mice. Reduces VCAM-1 mRNA levels in the lungs of LPS-stimulated mice (150 mg/kg/day)		Sundell *et al.*^86^
		*Clinical trials:* Atherosclerosis regression was observed with treatment, although this was not significantly different from placebo (300 mg/kg/day)		Tardif *et al.*^84^
Natural products	Halichlorine	*In vitro:* Selectively inhibits induction of VCAM-1 expression (IC_50_ = 7 µg/mL)	Efficacy has not been assessed *in vivo* or in clinical studies	Kuramoto *et al.*^87^ Trauner *et al.*^88^
		*In vitro:* Inhibits LPS-induced VCAM-1 expression and U937 monocyte adhesion to BAECs (10 µM)		Tsubosaka *et al.*^89^
	Curcumin	*In vitro:* Suppresses VCAM-1 expression and U937 adhesion IL-1β–stimulated HUVECs (10–20 µg/mL)	Conflicting results between studies suggest the need for further clinical testing	Chang *et al.*^90^
		*In vitro:* Suppresses VCAM-1 expression TNF-α–stimulated HUVECs (25 µg/mL)		Lee *et al.*^91^
		*In vitro:* Suppresses VCAM-1 expression in TNF-α–stimulated HUVECs (1 µM). Reduces U937 adhesion to stimulated HUVECs (0.1–1 µg/mL) *In vivo:* Decreases atherosclerotic lesion area and macrophage infiltrate number in aorta of *ApoE^-/−^* mice (0.2% w/w)		Coban *et al.*^92^
		*In vitro:* Reduces monocyte adhesion to and transmigration across TNF-α–stimulated HUVECs (0.5–1 µM)		Monfoulet *et al.*^93^
		*In vitro:* Decreases particulate matter-induced endothelial VCAM-1 expression in human microvascular ECs (10–50 µM)		Shi *et al.*^94^
		*In vivo:* Reduces atherosclerotic lesion severity in male New Zealand white rabbits (1–10 mg/kg/week)		Momtazi-Borojeni *et al.*^95^
		*In vivo:* Decreases aortic lesion area and neo-intimal formation. Reduces mRNA and protein expression of VCAM-1 (0.2% w/w)		Um *et al.*^96^
		Improves serum lipid profile in subjects with Type 2 diabetes (1000 mg/day plus 10 mg/day piperine)		Panahi *et al.*^97^
		Reduces aortic stiffness in young, obese men (500 mg/day)		Campbell *et al.*^98^
		*Clinical trial:* Improves endothelial function in healthy adults (50–200 mg/day)		Oliver *et al.*^99^
Peptide antagonists	CQIDSPC	*In vitro:* Inhibits Ramos cell adhesion to purified VCAM-1 (0.2–1.0 mM)	Efficacy has not been assessed *in vivo* or in clinical studies	Wang *et al*.^100^
	ILDV	*In vitro:* Inhibits MOLT-4 attachment to immobilized rsVCAM-1 and VCAM-1-transfected COS-1 cell monolayers. Reduces A375 melanoma cell spreading on immobilized rsVCAM-1 (1–3 mg/mL)		Makarem *et al*.^101^
		*Ex vivo:* Inhibits U937 accumulation in carotid arteries from *ApoE^−/−^* mice		Huo *et al*.^60^
Monoclonal antibodies	H6	*In vitro:* Inhibits U937 (0.1–10 µg/mL), CD4^+^ T-cell (1–100 µg/mL), and EoL-1 cell (0.1–10 µg/mL) adhesion to immobilized rsVCAM-1. Prevents U937 adhesion to and transmigration across TNF-α–stimulated HUVECs (0.01–10 µg/mL)	Efficacy has not been assessed *in vivo* or in clinical studies	Park *et al*.^102^
	7H	*In vitro:* Dose-dependently inhibits adhesion of U937 monocytes (0.1–10 µg/mL), CD4^+^ T-cells (1–100 µg/mL), and EoL-1 cells (0.1–10 µg/mL) to immobilized rsVCAM-1. Prevents U937 adhesion to and transmigration across TNF-α–stimulated HUVECs (0.01–10 µg/mL) and CD11b^+^ adhesion to MAECs (10 µg/mL) *In vivo:* Decreases atherosclerotic plaque area in aortic sinuses of *ApoE^−/−^* mice by specifically binding VCAM-1 (1–10 mg/kg)	Efficacy has not been assessed in clinical studies. Therapeutic efficacy may have been confounded by the production of anti-human murine antibodies against humanized 7H mAb	

Treatment concentration and time of therapeutic for specific experiments are provided in parentheses. COS-1, CV-1 Origin SV40; CQIDSPC, Cys-Gln-Ile-Asp-Ser-Pro-Cys; ILDV, Ile-Leu-Asp-Val.

## 4. Therapies that suppress VCAM-1 expression and signalling

Most VCAM-1-inhibiting anti-atherosclerotic agents focus on suppressing VCAM-1 expression and function by modulating upstream inflammatory mediators and signalling intermediaries. The rationale for this strategy draws back to the concept that pathological CAM expression during vascular inflammation is induced by the inflammatory gene expression programme encoded by innate immunity.^103^ Several regulatory molecules inherent to this response have been considered as potential therapeutic targets for treating inflammation, including cytokines, transcriptional regulators, and protein machinery.^104^ One of the most extensively studied of these in the context of atherosclerosis is the pro-inflammatory nuclear factor-κB (NF-κB) master regulator. NF-κB signalling activates multiple target genes associated with vascular inflammation and acts as a pivotal regulator in the pathophysiology of atherogenesis.^105,106^ The NF-κB dependence of VCAM-1 is evident on the gene level by the presence of two consensus NF-κB sites in the *VCAM1* promoter required for cytokine-induced expression.^107,108^ Various novel therapeutics modulating NF-κB activity have revealed how the inducible nature of VCAM-1 can be manipulated via precursor signalling pathways and regulatory mechanisms.^109^ Therefore, drug candidates targeting inflammatory induction of VCAM-1 expression and signalling remain an encouraging pharmacological strategy against atherosclerosis.

### 4.1 Proteasome inhibitors

The central role of proteasomes in modulating pro-inflammatory effector proteins has prompted studies into the therapeutic potential of proteasome inhibitors (PIs) for immunological disorders.^110^ The ubiquitin-proteasome system (UPS) mediates the activation of the NF-κB pathway through the proteasomal degradation of IκB kinase, which in turn regulates transcription of several pro-inflammatory molecules, including VCAM-1.^111^ Read *et al.*^66^ proposed disrupting the UPS as a potential therapeutic strategy to regulate aberrant CAM expression during vascular inflammation in an investigation of the PI, MG-132. This study demonstrated that MG-132 profoundly blocked cytokine-induced ICAM-1 and VCAM-1 surface expression and leucocyte transmigration in cultured human umbilical vein ECs (HUVECs). Shortly following this, Cobb *et al.*^63^ demonstrated that MG-101 PI inhibited VCAM-1 and ICAM-1 promoter activity and surface expression in stably transfected ECs and HUVECs without affecting the nuclear translocation of NF-κB, albeit to a lesser degree compared to MG-132. The therapeutic potential of PIs has since been evaluated in the context of several immunological and inflammatory diseases^112–114^ but has proven somewhat problematic in experimental models of atherosclerosis and other CVDs.

Contradictory findings suggesting differential effects of proteasome inhibition in the vascular environment have curbed enthusiasm for PIs as anti-atherosclerotic therapies.^64,115^ A study by Tummala *et al.*^64^ supported the atheroprotective potential of MG-101 and lactacystin PIs as VCAM-1–blocking agents by inhibiting angiotensin II-induced VCAM-1 mRNA accumulation and promoter transactivation via the NF-κB pathway in rat aortic smooth muscle cells (RASMCs) modelling arterial hypertension. However, Herrmann *et al.*^115^ contrarily found that chronic, low-dose administration of MLN-273 PI had a detrimental effect on oxidative stress and vascular injury in hypercholesterolemic pig models, measuring increases in VCAM-1 expression, monocyte infiltration, and coronary intimal thickness. An accompanying editorial by Fukai^116^ hypothesized that these inconsistencies could likely be attributed to multiple factors such as the stage of atherosclerosis, specific characteristics of the cell model, dose-dependency, and means of administration. The credibility of future studies will hinge on the proper articulation of these parameters within both cell and animal models in order to reach a definitive conclusion as to whether targeting the proteasome is beneficial or, in fact, detrimental to atherosclerosis.^117^

#### 4.1.1 Lactacystin

The ability of lactacystin to specifically and irreversibly bind the proteasome sparked research interest in its therapeutic potential for inflammatory diseases involving the UPS, such as atherosclerosis.^68^ In a study characterizing the role of endothelium-dependent mechanisms in pathological leucocyte transmigration, Allport *et al.*^65^ demonstrated that treatment with lactacystin inhibited surface expression of E-selectin, ICAM-1, and VCAM-1 and functionally suppressed inflammatory adhesion and transmigration of human neutrophils with HUVECs. Similarly, Dagia and Goetz^69^ demonstrated that lactacystin treatment significantly inhibited CAM expression and H670 myeloid cell adhesion to IL-1β- and tumour necrosis factor-α (TNF-α)–activated HUVECs. The therapeutic effects of lactacystin have also been observed in vascular models, observing that lactacystin treatment inhibits angiotensin II-induced VCAM-1 mRNA accumulation^64^ and neo-intimal formation^68^ in RASMCs. However, a more recent study by Simko *et al.*^67^ contrarily found that chronic administration of lactacystin induces hypertension in male Wistar rats, suggesting that long-term treatment would likely contribute to atherosclerosis. In light of these contradictory findings, more comprehensive studies on the molecular mechanisms of lactacystin in atherosclerosis will need to be conducted before progressing to clinical testing.

#### 4.1.2 Bortezomib

A particularly notable PI in clinical practice is bortezomib, which has been investigated for its anti-atherosclerotic potential following its success as an anti-cancer drug for multiple myeloma and mantle cell lymphoma.^118^ Hund *et al.*^119^ demonstrated that bortezomib treatment in combination with dimethyl fumarate down-regulated the VCAM-1 expression and functional adhesion of lymphocytes, warranting further pre-clinical study to investigate this anti-inflammatory activity. Wilck *et al.*^73^ found that administration of low-dose bortezomib significantly reduced VCAM-1 expression, macrophage infiltration, and early atherogenesis in *LDLR^−/−^* mice. However, this research was opposed in a later study by Ismawati *et al.*,^72^ which demonstrated that a short-term dosage regimen of bortezomib did not suppress atherosclerotic lesion formation in atherogenic Wistar rat models despite reducing VCAM-1 expression. Another concern is the occurrence of adverse CVD events that have been observed in existing clinical studies with multiple myeloma patients.^70^ Though these issues do not entirely rule out bortezomib as a potential anti-atherosclerotic drug candidate, it certainly reinforces the need for further research and systematic analysis in alternative *in vitro* and *in vivo* models before progressing with clinical studies.^71^

### 4.2 Antioxidants

Antioxidants, which can inhibit oxidative damage by scavenging-free radicals before they react with intra-cellular biomolecules, represent an attractive strategy for the treatment and prevention of atherosclerosis.^120^ Oxidative stress is a critical pathogenic contributor to atherosclerosis, most notably through oxidized LDLs and EC dysfunction.^121^ Specifically, the causal relationship between oxidative stress and aberrant VCAM-1 expression, along with the inherent role of ROS in VCAM-1 signalling, has implicated antioxidants as a potential avenue for anti-atherosclerotic therapeutics targeting monocyte infiltration. Marui *et al.*^74^ elucidated an antioxidant-sensitive transcriptional regulatory mechanism linking oxidative stress and VCAM-1 activity, which could be repressed by two antioxidants, pyrrolidine dithiocarbamate (PDTC) and N-acetylcysteine (NAC). This study observed that pre-treatment with PDTC or NAC blocked the induction of functional VCAM-1 protein expression through an NF-κB regulatory element in stimulated HUVEC models. Moreover, PDTC treatment markedly attenuated VCAM-1–mediated monocyte adhesion. Fruebis *et al.*^122^ later supported the notion that antioxidants reduce atherosclerosis by modulating the expression of VCAM-1 via oxidation-dependent signalling, demonstrating that probucol treatment and vitamin E supplementation in hypercholesterolaemic rabbit models significantly attenuated arterial VCAM-1 expression and atherogenesis *in vivo*.

However, gaining a better understanding of oxidative mechanisms in atherosclerosis has noticeably dampened enthusiasm for antioxidant therapies in recent years. Although there is a wide variety of literature discussing the effects of antioxidants in atherosclerosis and CVD, there is yet to be a solid consensus regarding the relative merits of antioxidant-based strategies as reliable anti-atherosclerotic therapies. A recent review by Cabezas *et al.*^123^ noted a lack of scientific data definitively correlating prophylactic and non-prophylactic antioxidant treatment to a significant decrease in atherosclerosis-associated mortality and comorbidities. From this, the authors concluded that the pharmacological mechanisms of antioxidants may likely be overwhelmed by other disease pathways involved in atherosclerosis. As such, while oxidative stress is a crucial factor in a range of disease pathologies, antioxidant therapy often fails to inhibit the progression of tissue injury once other factors become more dominant.^124^ This limitation presents a significant challenge for the therapeutic application of oxidation-targeted anti-atherosclerotic therapies and explains the recent lack of success of antioxidant drug candidates in clinical trials.^125^ Antioxidant therapies are continuing to be investigated despite these setbacks, albeit with a suggested shift in context to specific subsets of CVD patients exhibiting high levels of oxidative stress or deficient natural antioxidant defence systems.^126^

#### 4.2.1 Bilirubin

The potent antioxidant and anti-thrombotic activity of the tetrapyrrole pigment, bilirubin, has recently garnered its attention as a potential endothelial protective agent from oxidative stress during vascular inflammation and atherosclerosis.^77,127^ Mazzone *et al.*^77^ demonstrated that treatment with unconjugated bilirubin inhibited VCAM-1, ICAM-1, and E-selectin expression in HUVEC and murine heart endothelial immortalized cell (H5V) models, suggesting an anti-inflammatory mechanism of bilirubin in pathological cell adhesion processes mediated by CAMs. Additionally, Keshavan *et al.*^76^ found that high bilirubin concentrations blocked VCAM-1 activation of MMPs involved in endothelial junctional opening and lymphocyte transmigration in murine high endothelial venule-like (mHEv) cell models. Vogel *et al.*^78^ corroborated these findings *in vivo* by demonstrating that bilirubin treatment of *LDLR^−/−^* mice ameliorates atherosclerotic lesion formation by scavenging ROS signalling intermediaries and consequently disrupting VCAM-1 and ICAM-1-mediated leucocyte migration. Although the atheroprotective benefits of bilirubin are yet to be observed in clinical intervention studies, multiple observational analyses have observed an inverse relationship between elevated serum bilirubin levels and CVD incidence.^128–130^ Further exploration to decisively establish a direct causal relationship between bilirubin treatment and decreased CVD risk remains a research direction of interest for anti-atherosclerotic drug development.

#### 4.2.2 Probucol and succinobucol

Although traditionally employed as a cholesterol-lowering drug targeting LDL accumulation, probucol has more recently generated significant research attention for its antioxidant activities directly combatting the inflammatory processes underlying atherogenesis.^131^ In an investigation of probucol’s method of action *in vitro*, Zapolska-Downar *et al.*^83^ attributed its anti-atherogenic effects partly to the selective down-regulation of VCAM-1 expression. The authors demonstrated that treating cytokine-stimulated HUVECs with probucol reduced VCAM-1 mRNA accumulation and protein surface expression, consequently decreasing the adherence of peripheral blood mononuclear leucocytes. The VCAM-1 inhibitory and atheroprotective effects of probucol *in vivo* have also been supported in several animal models, including apolipoprotein E-deficient (*ApoE^−/−^*) mice^82^ and Watanabe heritable hyperlipidaemic rabbits.^80,81^ Probucol studies have progressed to clinical trials. Yamashita *et al.*^79^ recently investigated whether probucol treatment combined with other conventional lipid-lowering therapies (LLTs) could prevent cerebrovascular and CVD events in Japanese patients with coronary artery disease and hyperlipidaemia. However, while the researchers observed that probucol treatment decreased the incidence of CVD events compared to LLTs alone, the failure of this study to produce a statistically significant association prompts the need for further examination and analysis of probucol in the clinical context.

In recent years, significant research attention has been diverted to the metabolically stable monosuccinic acid ester of probucol, succinobucol (AGI-1067), as a potential treatment option for the secondary prevention of atherosclerosis. Succinobucol was designed to retain the favourable antioxidant and lipid-lowering activities of probucol while improving its hydrophilicity, cellular uptake, and retention.^86,132^ Kunsch *et al.*^85^ elucidated the antioxidant mechanisms of succinobucol *in vitro* using a variety of EC lines, suggesting that succinobucol treatment inhibited inducible VCAM-1 expression by inhibiting ROS-sensitive gene expression independently of the NF-κB master regulator. Sundell *et al.*^86^ reported that succinobucol treatment inhibited VCAM-1 and monocyte chemoattractant protein-1 (MCP-1) mRNA in lipopolysaccharide (LPS)-stimulated mice and decreased atherosclerotic lesion formation in *LDLR^−/−^* and *ApoE^−/−^* mice. Following encouraging results *in vitro* and *in vivo*, the Canadian Antioxidant Restenosis Trial conducted by Tardif *et al.*^84^ demonstrated atherosclerosis regression with succinobucol treatment in patients after percutaneous coronary intervention; however, this was not significantly different from placebo. Investigations into succinobucol have continued despite this somewhat unfulfilling outcome, albeit following a shift in focus to atherogenic processes outside of VCAM-1-mediated cell recruitment.^133^

### 4.3 Other natural products

Due to the challenges that arise in balancing the safety and efficacy of conventional pharmaceuticals, natural drug derivatives are becoming progressively more appealing for the design and development of anti-atherosclerotic drug candidates. The chronic nature of atherosclerosis means that, more often than not, anti-atherosclerotic treatment regimens must be long-term. Taking into account the risks of tachyphylaxis and toxicity that arise when administering conventional anti-atherosclerotic medications for extended periods, researchers must now consider natural alternative platforms that can be therapeutically feasible in the long term.^134^ Current curiosities in traditional medicines have opened the door to a whole reservoir of marine-, plant-, and microbial-derived VCAM-1 inhibitors that might display therapeutic potential against atherosclerosis.^135^ However, research into alternative natural remedies for atherosclerosis remains highly experimental, with only a minority of these novel therapeutics gaining the research momentum to progress into the clinical testing phase. Nonetheless, natural products remain a field of great interest within the research scope of atherosclerosis, with a recent emergence of investigative studies documenting the atheroprotective potential of novel bioactive compounds and current natural drug candidates.

#### 4.3.1 Halichlorine

The significant VCAM-1-inhibitory activity of halichlorine, a novel alkaloid isolated from the black marine sponge *Halichondria okadai* Kadota, has implicated it as a potential treatment for inflammatory diseases such as atherosclerosis.^136,137^ Halichlorine first aroused research interest after being demonstrated to selectively inhibit the induction of VCAM-1, prompting investigations into the compound as a prospective inhibitor of monocyte infiltration during atherosclerosis.^87,88^ Tsubosaka *et al.*^89^ examined the anti-atherosclerotic mechanism of halichlorine *in vitro*, demonstrating that pre-treatment of LPS-stimulated bovine aortic ECs (BAECs) with halichlorine suppressed VCAM-1, ICAM-1, and E-selectin surface expression as well as U937 monocyte adhesion without detrimental effects to cell viability. The authors attributed this to the suppression of NF-κB p65 translocation, possibly via IκB inhibition or NF-κB phosphorylation, which would consequently down-regulate VCAM-1 expression and function. Halichlorine is yet to be tested *in vivo* or in clinical studies; therefore, further analyses are necessary before reaching a definitive conclusion on its therapeutic mechanism and potential for atherosclerosis.

#### 4.3.2 Curcumin

The complementary lipid-lowering and antioxidant effects of curcumin, a bioactive polyphenolic compound obtained from the turmeric rhizomes of *Curcuma longa*, have recently commanded its attention as a prospective natural remedy for atherosclerosis.^138,139^ Curcumin has been well-characterized for its abilities to modulate pro-atherogenic signalling *in vitro*. Most notably, it prevents pathological VCAM-1 overexpression and leucocyte recruitment via the immunoblockade of antecedent NF-κB- and Toll-like receptor 4 –dependent pathways.^90–94^ This atheroprotective potential has been corroborated *in vivo* with various animal models of experimental atherosclerosis.^95,96,140^ In particular, Um *et al.*^96^ observed that curcumin treatment decreased mRNA and protein expression of ICAM-1, VCAM-1, P-selectin, and MCP-1 and resultant monocyte adhesion at atheroprone arterial regions in hypercholesterolaemic New Zealand white rabbits. Clinical trials of curcumin treatment have so far shown promise, with indicators of decreased CVD risk observed in diabetic,^97^ obese,^98^ and healthy^99^ study populations. However, a meta-analysis by Sahebkar^141^ demonstrated that curcumin supplementation had no appreciable cardio-protective effect on blood lipid levels. These conflicting findings mean that additional studies, especially large-scale randomized trials, will be required to characterize the full potential of curcumin in atherosclerosis in the clinical setting,^138^ especially following the discovery of new nano-formulations that improve oral bioavailability.^142,143^

### 4.4 Statins

It is important to note that some current LLTs, most notably statins, exhibit some degree of anti-atherogenic and VCAM-1–antagonizing activity. The primary mechanism of statins is that they lower LDL levels by competitively inhibiting HMG-CoA reductase, a rate-limiting enzyme associated with cholesterol biosynthesis.^144^ Aside from lowering cholesterol, statins have also been observed to shift the immune response to an anti-inflammatory expression programme by suppressing signalling pathways such as NF-κB.^145^ From this, it has been proposed that statins can potentially ameliorate atherosclerotic lesion progression by suppressing the vascular expression of CAMs and thus inhibiting monocyte recruitment.^146^ In the context of VCAM-1 specifically, however, findings have been somewhat discrepant, with studies reporting both the suppression^147,148^ and superinduction^149,150^ of endothelial VCAM-1 expression with statin treatment. The pleiotropic effects of statins on inflammation also present a double-edged sword, as the potential for disrupting immune homeostasis with long-term treatment carries the risk of inducing adverse autoimmune responses, such as statin-associated muscle toxicity.^151^ As such, although statins remain a first-line treatment for atherosclerosis and CVD, researchers continue to investigate alternative pharmacological strategies that may allow more streamlined and effective targeting of the specific inflammatory processes underlying atherogenesis.

## 5. Therapies that directly inhibit the VCAM-1/VLA-4 interaction

In general, the design of VCAM-1–inhibiting agents has not so much centred on the direct neutralization of VCAM-1 but rather on suppressing its expression as a symptom of the larger vascular inflammatory phenotype. So far, this review has discussed several anti-atherosclerotic therapies that pharmacologically modulate pleiotropic regulatory molecules associated with inflammation to disrupt the induction and expression of VCAM-1. This section will cover a selection of novel therapeutics that directly interfere with the VCAM-1/VLA-4 interaction involved in monocyte-endothelial attachment and infiltration.

While inhibiting inflammatory mediators has the advantage of blocking multiple effector proteins that may be dysregulated during inflammation, ubiquitously blocking an entire expression profile of immune mediators may carry disastrous repercussions for normal immunological function. For example, the anti-inflammatory mechanism of canakinumab, a monoclonal antibody (mAb) targeting the master IL-1β cytokine, against atherosclerosis has elicited a mixed reception due to its adverse association with leucopoenia and a higher incidence of fatal infections.^152,153^ In noticing the side effects of targeting broadly acting inflammatory mediators both *in vivo* and in clinical trials, researchers have come to realize that the large-scale inhibition of multiple CAM families on the vascular endothelium may not be a therapeutically feasible strategy.^16^ Instead, the ideal adhesion-based treatment for atherosclerosis should limit cell extravasation in such a way that suppresses pathological leucocyte infiltration while interfering with normal physiological leucocyte trafficking as little as possible. This reasoning has driven the transition towards specificity, with researchers designing highly selective anti-atherosclerotic novel therapeutics that directly target the VCAM-1/VLA-4 interaction with minimal effects on other CAMs and inflammatory molecules.

### 5.1 Peptide antagonists

Novel peptide antagonists able to directly target VCAM-1 involvement in leucocyte recruitment hold considerable promise as experimental therapies for atherosclerosis.^154^ Peptide ligands represent an attractive therapeutic strategy due to their low molecular weight, convenient handling and storage, and high specificity and affinity towards biological targets.^155^ Perhaps the most alluring characteristic of peptide-based therapeutics is their inherent biocompatibility and biodegradability *in vivo*, which allows them to overcome barriers in clinical translation that traditional pharmaceuticals may face.^156^ Novel peptides can be identified using a variety of complementary approaches, including structure-based computational docking methods, phage display, and synthetic peptide screening.^154,157,158^ Since first characterizing the three-dimensional structure of VCAM-1 using X-ray diffraction and crystallographic analysis,^159,160^ researchers have unlocked a wide arsenal of complementary peptide and peptidomimetic antagonists for selective targeting and discrimination of atherosclerotic lesions on the vascular endothelium. This vast reservoir of potential peptide drug candidates, combined with the rapid advancement of biotechnologies and synthesis platforms, delineates an optimistic future for VCAM-1 peptide antagonists in atherosclerosis.

Although peptide-based novel therapeutics are rapidly gaining traction as anti-atherosclerotic drug candidates, significant logistical challenges remain to be addressed. Multiple studies have proposed promising anti-inflammatory peptide antagonists, yet few peptide-based immunotherapies have progressed to clinical testing. This apparent roadblock in drug development can be attributed to the inherent physicochemical drawbacks of peptide molecules, most notably their increased proteolytic instability compared to small molecule and antibody therapeutics.^161^ The presence of multiple peptidases and peptide-clearing excretory mechanisms within the human physiological environment leads to short plasma half-life and low oral bioavailability, which are undesirable pharmacokinetic features for drug candidates.^162^ However, the continual refinement of peptide synthesis and modification techniques may prove these limitations to be only temporary. Novel rational drug design and phage display technologies combined with chemical and genetic modification techniques allow for the large-scale production of peptide and peptidomimetic drug candidates with enhanced stability and physiological activity.^163^ Nonetheless, further investigations of VCAM-1 protein structure and interactions during atherosclerosis are necessary to optimize the pharmacodynamic properties of future peptide therapies.

#### 5.1.1 CQIDSPC peptide

The cyclic Cys-Gln-Ile-Asp-Ser-Pro-Cys (CQIDSPC) peptide first designed and synthesized by Wang *et al.*^100^ has been shown to significantly inhibit monocyte transmigration by inhibiting the VCAM-1/VLA-4 interaction, serving as a base model for future VCAM-1–targeted peptide therapeutics. Following crystallographic studies characterizing the three-dimensional structure of the N-terminal dimeric fragment of VCAM-1,^159,160^ Wang *et al.*^100^ discovered that several residues critical for binding VLA-4 reside within the projecting C-D loop of Domain 1. The authors subsequently demonstrated that treatment with a cyclic CQIDSPC peptide mimicking the C-D loop inhibited adhesions between purified VCAM-1 and integrins on Ramos cells in a peptide binding assay compared to a linear QIDSP peptide control. Although this investigation exhibited that CQIDSPC could effectively block the pathological cell adhesion between VCAM-1 and VLA-4, research into this specific peptide has not progressed beyond *in vitro* study. The results of Wang *et al.*’s^100^ study, rather, aim to serve as a model for designing and developing future peptide therapeutics that mimic the loop structures of VCAM-1 to competitively inhibit VCAM-1–mediated cell recruitment during inflammation.

#### 5.1.2 ILDV peptide

**Figure 5:**
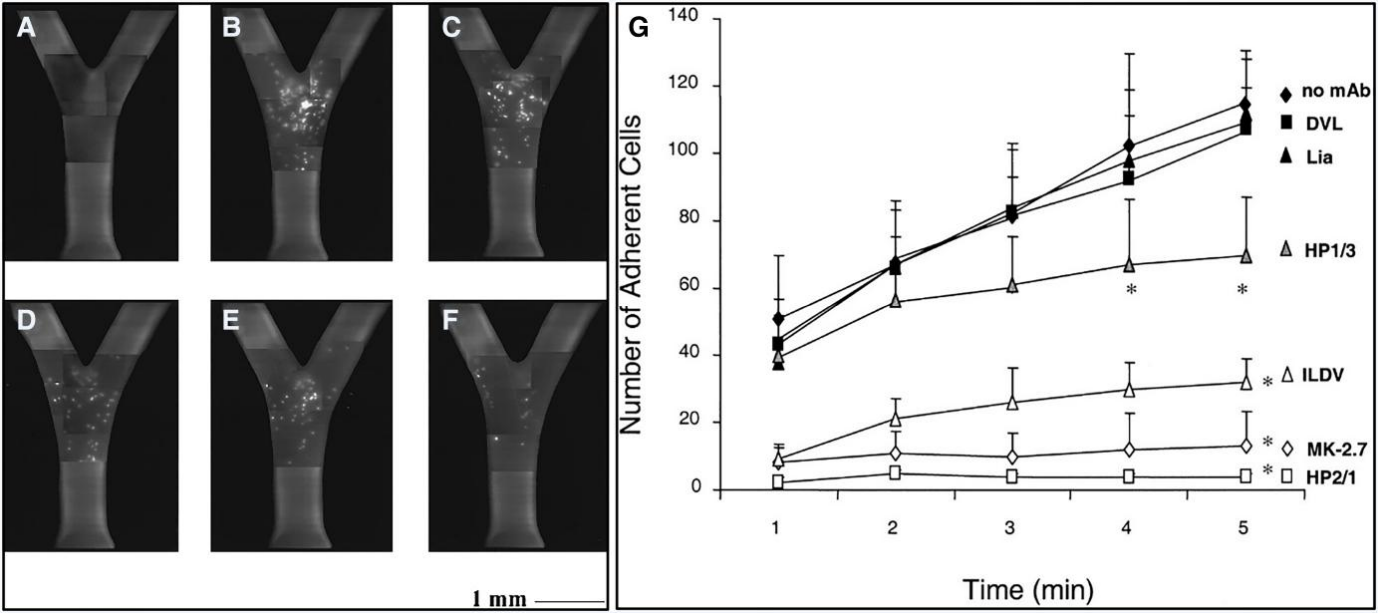
Comparing monocyte attachment following selected peptide- and antibody-based treatments targeting the VCAM-1/VLA-4 interaction. U937 adhesion to isolated perfused carotid arteries of *ApoE^−/−^* mice was visualized using composite epifluorescence videomicrography. (*A–F*) Treatment with (*A*) 3-(N-morpholino)propanesulfonic acid (MOPS) buffered salt solution, (*B*) non-blocking control mAb Lia, (*C*) anti-VLA-4 mAb HP1/3, (*D*) anti-VCAM-1 mAb MK-2.7, (*E*) ILDV peptide, and (*F*) anti-VLA-4 mAb HP1/2. (*G*) The accumulation of monocytes was subsequently quantified and plotted. The graphed data illustrate that ILDV peptide, MK-2.7 mAb, and HP2/1 mAb significantly reduced monocyte accumulation compared to MOPS and non-blocking mAb Lia controls (Huo *et al*.^60^).

The VLA-4–binding Ile-Leu-Asp-Val (ILDV) moiety is an another notable peptide drug candidate that can competitively inhibit VCAM-1–mediated cell recruitment during atherosclerosis. This sequence is based on the minimal binding epitopes contained within VLA-4–binding ligands, specifically LDV in fibronectin connecting segment-1 (CS-1)^164^ and the homologous IDS tripeptide in VCAM-1.^165^ Makarem *et al.*^101^ demonstrated the VCAM-1–antagonizing activity of an ILDV-containing CS-1 peptide that effectively inhibited MOLT-4 T-lymphoblastic leukaemia cell attachment to immobilized recombinant soluble VCAM-1 (rsVCAM-1), MOLT-4 attachment to monolayers of VCAM-1–transfected CV-1 Origin SV40 cells, and A375 melanoma cell spreading. Huo *et al.*^60^ later corroborated this in an *ex vivo* model of atherosclerosis by observing the effects of peptide treatment on U937 monocyte perfusion through carotid arteries obtained from *ApoE^−/−^* mice (*Figure 5*). This study found that ILDV-containing peptide treatment dramatically reduced mononuclear cell accumulation and therefore could present an effective strategy for attenuating monocyte rolling and adhesion during early atherosclerosis. Although research attention has shifted in favour of other VCAM-1–directed peptides, the evidence accumulated from ILDV studies holds significant implications for designing specific anti-inflammatory peptide therapeutics directed at the VCAM-1/VLA-4 interaction.

### 5.2 Monoclonal antibodies

While the majority of mAbs considered as potential anti-atherosclerotic agents has so far been lipid- or cytokine-directed, the recent shift in favour of more precisely targeted therapeutic strategies has driven investigations into anti-VCAM-1 mAbs. Soriano *et al.*^50^ aptly summarized the appeal of selective VCAM-1 mAbs over broad-spectrum CAM inhibitors in a colitic mouse model, proposing that exclusively inhibiting VCAM-1 could present a viable strategy for treating inflammation while still preserving physiological leucocyte trafficking. Research into VCAM-1 mAbs as novel anti-inflammatory agents has been conducted in several immunological disease models involving VCAM-1 overexpression, such as asthma^49,166,167^ and colitis.^50,168,169^ While the role of VCAM-1 has been well-characterized in atherosclerosis, studies into the anti-atherosclerotic potential of VCAM-1 mAbs remain extremely limited. The localization of VCAM-1 mAbs to the inflamed vascular endothelium in atherosclerotic lesions has seen VCAM-1 widely utilized as a target for *in vivo* imaging^170,171^ and drug delivery^172,173^ to atheromatous lesions. However, these applications have overshadowed its therapeutic potential for inhibiting pathological monocyte infiltration. Regardless, investigating the functional inhibition of VCAM-1 by specific mAbs has yielded promising results,^60,102^ warranting further pre-clinical exploration in atherosclerotic disease models.

Despite the therapeutic promise of mAbs for treating atherosclerosis, some logistical challenges remain regarding their design and development. The need for sophisticated eukaryotic machinery to produce active mAbs means they can be expensive to manufacture, even more so when considering the large amount that generally must be administered to achieve clinical efficacy.^174^ Like all biotherapeutics, mAb can also only be administered intravenously or subcutaneously as lyophilized and liquid-based formulations to achieve maximum bioavailability in a minimal volume.^175^ The inherent complexity of mAbs can also present obstacles during clinical translation, particularly when attempting to elucidate drug mechanisms once injected in human subjects.^176^ Limitations exist not only in mAbs themselves, but in the current methods we use to test them—particularly regarding the gap in complexity between conventional *in vitro* and *in vivo* models.^177^ This gap has stimulated the demand for improved *in vitro* technologies that can better mimic the human physiological environment during atherosclerosis as a better platform for evaluating mAbs, especially fully humanized mAbs. Rather than being viewed as dead-ends, these challenges have driven the advancement and improvement of current biotechnologies. As such, antibody-based therapeutics remain wholly one of the most promising pharmaceutical fields against atherosclerosis.

#### 5.2.1 HD101 mAbs (H6 and 7h)

An investigative study of the two fully humanized HD101 mAbs, H6 and 7H, by Park *et al.*^102^ remains the most comprehensive evaluation of anti-VCAM-1 mAbs in atherosclerotic disease models to date. This study characterized the anti-atherosclerotic mechanism of VCAM-1 mAbs in suppressing monocyte recruitment and atherosclerotic plaque progression both *in vitro* and *in vivo*. The authors demonstrated that both H6 and 7H were able to effectively inhibit the adhesion of U937 monocytes, CD4^+^ T-cells, and eosinophilic leukaemic (EoL-1) cells to immobilized rsVCAM-1, as well as the adhesion and transmigration of U937 monocytes with TNF-α–stimulated HUVECs. However, while both mAbs bound human VCAM-1, only 7H could bind murine VCAM-1 for functional evaluation *in vivo*. 7H was found to significantly inhibit the adhesion of CD11b^+^ monocytes to mouse aortic ECs (MAECs) and decrease atherosclerotic plaque area in the aortic sinus of *ApoE^−/−^* mice fed a Western-type diet. While these results were cause for optimism, Park *et al.*^102^ acknowledged the limitations of mouse models for testing humanized mAbs, suggesting that anti-human murine antibodies may have confounded the therapeutic efficacy of 7H against atherosclerosis. The favourable results of H6 and 7H aptly represent the therapeutic potential of VCAM-1–directed mAbs in ameliorating atherosclerosis. Furthermore, they also exemplify the demand to improve *in vitro* technologies for future humanized mAbs that may be explored in pre-clinical testing.

## 6. Perspectives and conclusions

Although the role of VCAM-1 has been widely justified as a contributor to the inflammatory mechanisms underlying atherosclerosis, it is only relatively recently that research into VCAM-1–directed anti-atherosclerotic therapies has gained considerable momentum. VCAM-1 is but one of a wide assortment of effector proteins encoded by the pathological gene expression profile of activated ECs during vascular inflammation. As such, drug candidates specifically targeting VCAM-1 have long been drowned out in favour of cytokine- and transcriptional regulator-targeted immunomodulatory therapies that can ubiquitously suppress multiple pro-inflammatory gene products. However, various pre-clinical and clinical studies have demonstrated that these broadly acting immune modulators have their own shortcomings, evident from adverse immune reactions and inconsistent effectiveness for novel VCAM-1–inhibiting PIs and antioxidants. As a result, highly specific peptide- and antibody-based therapeutics that selectively inhibit the VCAM-1/VLA-4 interaction between vascular ECs and circulating monocytes have recently emerged as a promising avenue for adhesion-based anti-atherosclerotic therapies. While therapeutic peptides and antibodies still face logistical challenges regarding drug delivery and clinical translation, the rapid advancement of biotechnologies and drug design platforms has seen these obstacles as incentive for further systematic analysis rather than absolute roadblocks.

One of the greatest dangers when modelling multi-factorial disease pathologies such as atherosclerosis is over-simplification, which remains a persisting challenge in evaluating the therapeutic efficacy of various anti-atherosclerotic novel therapeutics. For example, PI drug candidates have demonstrated differential, even contradictory, effects between studies that cannot be attributed solely to suppressing NF-κB activity and consequent VCAM-1 expression. It is entirely possible that these discrepancies draw back to the failure to adequately address and account for the complex interplay between signalling pathways and oxidative stressors during atherogenesis. The failure of several antioxidants to produce statistically significant results in clinical trials also reflects how antioxidant effects are overshadowed by other inflammatory mechanisms in more advanced stages of atherosclerosis. The primary solution proposed for this is to improve pre-clinical atherosclerotic disease models to better mimic the multi-faceted nature of atherosclerosis, along with the physiological conditions of the human vascular environment. As such, it is necessary to develop better *in vitro* models, to fully articulate the complex interactions and mechanisms by which these therapies protect against atherosclerosis before progressing with further pre-clinical and clinical studies.

When attempting to treating localized pathologies such as atherosclerosis, systemically administered drugs may provoke non-specific side effects or alternatively, fail to elicit a sufficient response to achieve any significant therapeutic benefit.^178^ This limitation might explain the failure of several clinical trials assessing novel VCAM-1–inhibiting drug candidates, which has prompted researchers to look into new technologies for improving drug delivery and specificity. In particular, the passive diffusion and endocytosis properties of nanoparticles has seen them explored as precisely targeted carriers for anti-atherosclerotic medications.^179^ The integration of VCAM-1-targeting drugs into functionalized nano-systems presents a hopeful strategy, as can be seen from the therapeutic promise of curcumin nano-formulations in pre-clinical testing.^142,143^ The major advantage of nano-formulated drugs is that they can be delivered specifically to overexpressed VCAM-1 on the inflamed endothelium and therefore should be more robust to changes in inflammatory signalling and gene expression as atherosclerosis progresses. This notion is supported by the fact that, while many current anti-atherosclerotic drug candidates are only efficacious at specific stages of atherogenesis, novel VCAM-1–targeting nanoparticle systems have demonstrated therapeutic efficacy across early-, mid-, and late-stage plaque development.^180,181^

Finding the optimal balance between efficacy and safety will always present a challenge when developing immunomodulatory drugs for complex disease pathologies such as atherosclerosis. With this in mind, the inducible and localized nature of VCAM-1 expression presents its greatest asset as a therapeutic target in atherosclerosis, allowing researchers to precisely focus drug activity to sites of vascular inflammation. The literature supports the atheroprotective effects of pharmacologically inhibiting VCAM-1 through various drug mechanisms, which warrants further investigation into VCAM-1–directed strategies for combatting atherosclerosis. Future research should be directed towards designing and developing novel agents specifically antagonizing the VCAM-1/VLA-4 interaction to optimally suppress inflammatory cell recruitment at the receptor-ligand level. Furthermore, with the continued development and refinement of novel VCAM-1–antagonizing agents and VCAM-1–targeting nano-systems, the capabilities of anti-VCAM-1 agents as anti-inflammatory drug platforms will likely only strengthen in the near future.
